# Bird community responses to urbanization and vegetation parameters across the city of Salzburg, Austria

**DOI:** 10.1007/s11252-025-01738-y

**Published:** 2025-05-28

**Authors:** Beate A. Apfelbeck, Marina Navalpotro Buscail, Anna Sommer, Jana S. Petermann

**Affiliations:** 1 Department of Environment and Biodiversity, University of Salzburg, Hellbrunnerstrasse 34, 5020 Salzburg, Austria; 2 Ecology Department, Biology Faculty, University of Barcelona, Diagonal, 643, 08028 Barcelona, Spain

**Keywords:** Urban ecology, Bird species richness, Tree species richness, Bird community, Urban matrix

## Abstract

Urbanization continues to be a major threat to biodiversity. The knowledge of local and site characteristics that influence species diversity within cities is of importance to design and manage urban spaces that promote biodiversity and ecosystem services. While the characteristics of urban green spaces for biodiversity have been studied in detail, the urban matrix has received less attention. We, therefore, studied the role of sampling site and local-scale parameters at different urbanization intensities on bird and tree communities during the breeding season across the city of Salzburg encompassing the heterogeneity of the urban landscape. We found more exotic than native tree species, however areas with many trees, were dominated by native tree species. Although building cover negatively related to bird species richness at all scales, bird species richness increased with the amount of grass cover, the number of trees and the number of native tree species at the smallest, i.e. sampling site, scale. Building cover and the number of trees in a sampling site also influenced bird community composition. At larger scales, land cover (i.e., building cover and green urban area) was more strongly related to bird community composition than sampling site characteristics. Relationships with community composition can partly be explained by species-specific responses as the occurrence of different species was associated with different sampling site characteristics. Our study shows the importance of unsealed soil, i.e. grass cover, and native trees on bird communities within cities, but different species may require different configurations of these habitat parameters. Furthermore, also within the urban matrix site characteristics can promote avian diversity even in areas with high building cover.

**Supplementary Information:**

The online version contains supplementary material available at 10.1007/s11252-025-01738-y.

## Introduction

Land-use change and intensification for agriculture and settlements are major drivers of global biodiversity loss (Maxwell et al. 2016; Semenchuk et al. 2022). As human-modified areas are dominating the earth´s surface, it is increasingly important to examine their role for biodiversity conservation (Franklin and Lindenmayer 2009). For example, growing human urban populations are resulting in larger and denser cities (Ritchie and Rose 2018; Teller 2021). At the same time, despite their highly modified state, many animal and plant species are found in urban areas (Aronson et al. 2014; Callaghan et al. 2019). In addition, urban biodiversity also fulfils a number of important ecosystem services, such as water retention and air filtration, and contributes to the wellbeing and happiness of the urban human population (Schwarz et al. 2017). Thus, as urban biodiversity is relevant for animal conservation and healthy urban human populations, related research is now gaining interest from ecologists, conservationists, and urban planners (Apfelbeck et al. 2020).

A focus of many studies on urban biodiversity have been birds, which frequently occur in urban areas (Sweet et al. 2022), are easy to observe by both researchers and citizens and contribute to our life-satisfaction (Methorst et al. 2021). Within cities, the highest bird species richness is often found in vegetated, wooded, and wetland areas (Ferenc et al. 2016; Leveau et al. 2020). Therefore, until recently, much of the attention of urban ecologists has focused on remnant semi-natural areas, such as urban woodlands or meadows, or managed green spaces within the urban landscape, such as parks or cemeteries (Nielsen et al. 2014; Kowarik et al. 2016). In addition, private residential gardens have been recognized as important wildlife refuge because of their substantial combined size (Goddard et al. 2010). Furthermore, recent research shows that urban areas outside of these green spaces, i.e. the urban matrix, which includes for example public squares and roadsides, can also serve as habitat for wildlife given the presence of vegetated elements (Mühlbauer et al. 2021). The decline of species that were considered well adapted to the urban environment (Rosenberg et al. 2019; Mohring et al. 2021), the densification of cities and resulting lack of green spaces, emphasizes our need to understand which characteristics of the urban matrix promote wildlife.

Thus, to increase or at least maintain urban avian biodiversity it is necessary to determine which site and local-scale parameters contribute to bird diversity within urban areas (Ferenc et al. 2014). At the site-scale, i.e. at the smallest spatial scale, where birds breed and forage, it has been shown that the size and proportion of green, i.e. the amount of vegetated surface, in a site is a major determinant of bird species richness (Beninde et al. 2015; Fairbairn et al. 2024), while increasingly sealed areas and high building density negatively affect bird communities (Fontana et al. 2011; Morelli et al. 2024). In addition, urban spaces possessing different vegetation elements, i.e., grass, shrubs, and trees, can be home to a variety of birds (Belaire et al. 2014; Mühlbauer et al. 2021). Although bird species richness usually increases with the amount of vegetation in a site, species composition may vary depending on the presence and amount of specific vegetation parameters (Dale 2018; Mühlbauer et al. 2024). Furthermore, land cover, such as building density or forest cover, at a local scale, i.e. in the surroundings of a site, can affect bird community composition as well (Ferenc et al. 2014; van Heezik and Adams 2016).

Trees are a major component of urban green and tree cover, or tree density have been shown to positively affect urban birds in a variety of different urban spaces (Beninde et al. 2015; Lerman et al. 2021; Mühlbauer et al. 2021). Although other vegetation structures, such as shrub or grass cover, also positively correlate with the abundance of some bird species (Mühlbauer et al. 2024), trees often have a stronger influence on bird species richness (Beninde et al. 2015). While tree cover has a general positive effect on birds, tree community composition, such as the proportion of native versus exotic trees, matters for bird species richness, composition and abundance (Fontana et al. 2011; Narango et al. 2018). In addition, tree species richness can contribute positively to bird species richness and abundance (Ferenc et al. 2014; da Silva et al. 2021; Fairbairn et al. 2024). Trees also play a major role for bird species richness outside common green spaces. For example, street trees as part of avenues or planted individually, can provide valuable habitat for birds (Strohbach et al. 2013; Barth et al. 2015; Pena et al. 2017; Le Roux et al. 2018; Wood and Esaian 2020).

Tree species composition within cities varies significantly from forests in the surrounding areas, usually comprising a higher richness and diversity of trees, because of the presence of many non-native trees (Nielsen et al. 2014; Augustinus et al. 2024). In addition, trees are not uniformly distributed across the urban landscape and tree species composition varies with urban land use type, building cover and socioeconomic factors (Bourne and Conway 2014; Padullés Cubino and Retana 2023). Plant communities in cities are strongly influenced by human actions, i.e. through landscaping and gardening choices, and plants have been exchanged globally, explaining the high proportion of exotic plants within cities (La Sorte et al. 2007). Given that trees are a crucial part of urban biodiversity, a key component of green infrastructure, and an important landscaping element in cities, it is essential to understand their role for urban biodiversity and the factors influencing tree community composition.

Although the importance of urban biodiversity in the urban matrix has been recognized (Apfelbeck et al. 2020), the majority of studies so far has been restricted to urban green spaces, i.e. semi-natural areas, parks, cemeteries or gardens. In this study we, therefore, investigated the role of sampling site (50-m radius plots) and local-scale parameters (200-m and 500-m radius plots) at different urbanization intensities on bird and tree communities during the breeding season across the city of Salzburg encompassing the entire heterogeneity of the urban landscape. As birds are strongly influenced by the presence of trees in urban areas, and trees are a major landscape element, i.e. their presence in the urban environment is mainly a result of human decisions, we assessed the relationship between urbanization and richness and composition for birds and trees. We assessed different vegetation elements as well as building cover and impervious density at each sampling site and determined building cover and impervious density as well as the amount of different land-cover types, i.e., arable land, forest, and urban green within the surroundings of each sampling site (i.e., at the local scale). Similar to studies limited to urban green spaces we hypothesized that sampling site-scale vegetation parameters have a stronger influence on bird species richness and composition than land-cover in the surroundings of a sampling site. Furthermore, we hypothesized that tree species richness has a positive influence on bird species richness, which we expected to be mainly driven by native trees, not exotic ones. In addition, we hypothesized that tree species origin (i.e., native or exotic) affects bird species composition. Furthermore, for some bird species that occurred with sufficient frequency, we also determined whether characteristics of the sampling site influenced their occurrence and expected that responses of individual species may differ from relationships found for overall species richness because of different habitat needs (Mühlbauer et al. 2024). Finally, we also determined whether urban tree species richness and composition are influenced by sampling site and local-scale parameters.

## Material and Methods

### Study area

**Fig. 1:**
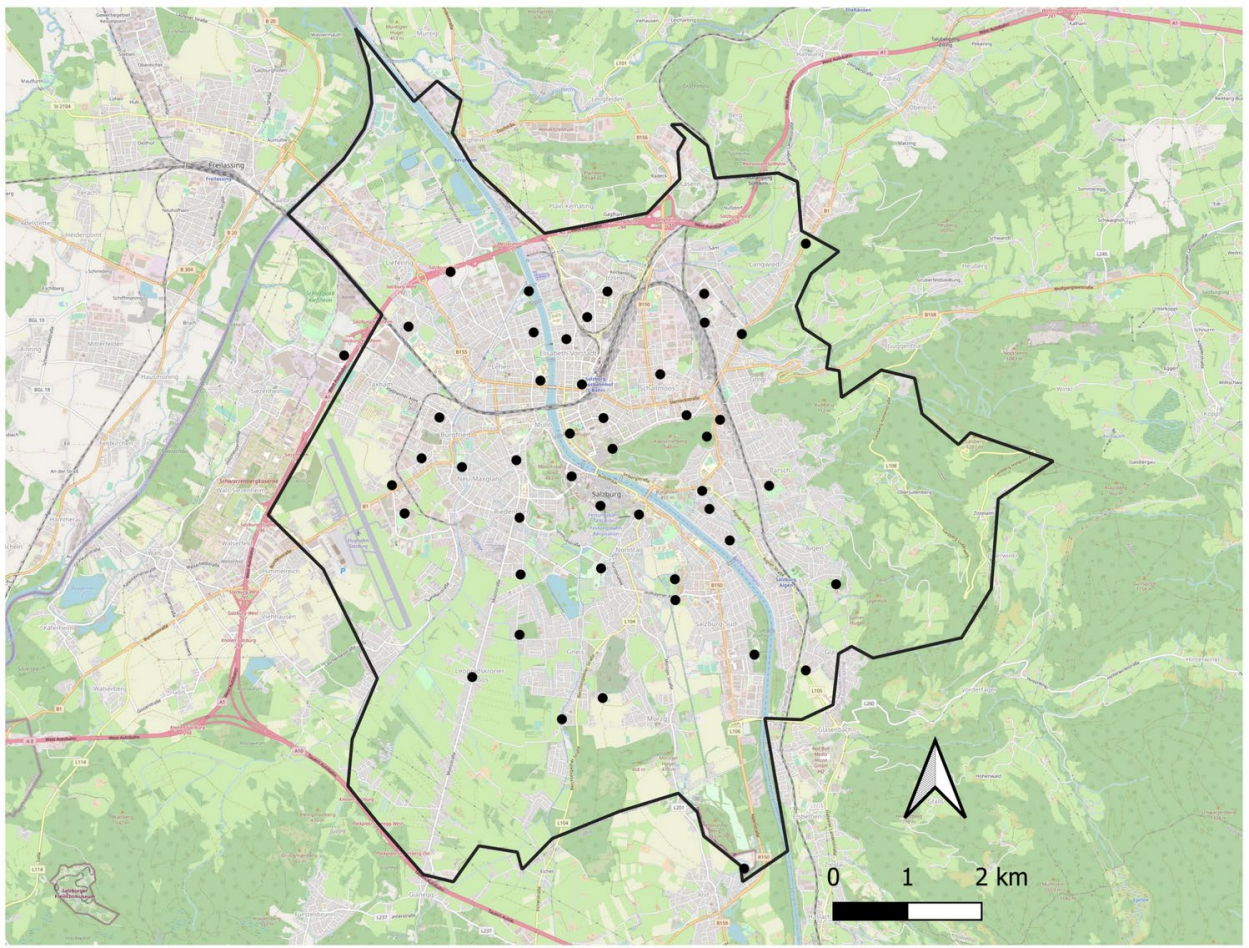
Map of the city of Salzburg showing the 48 sampling points of the study, created with QGIS 3.22.11. Data provided by OpenStreetMap. The city border (black line) was prepared using European Union’s Copernicus Land Monitoring Service (https://land.copernicus.eu/local/urban-atlas/urban-atlas-2018)

The study was carried out in Salzburg (47° 47′ 57.88"N, 13° 02′ 38.36"E, 424 m asl), the fourth largest city in Austria with about 155,000 inhabitants, located in the north-west of the country. The city of Salzburg extends over an area of 65.7 km^2^, of which 17.5 km^2^ are arable land, 11.9 km^2^ residential gardens, 10.7 km^2^ forest and 2.4 km^2^ waterbodies (Statistik_Austria 2020). The city of Salzburg also includes 10 larger urban parks (9 of them with a size between 20,000–80,000 m^2^ and one with a size of 500,000 m^2^). In addition to forests within the city boundaries, about 22,000 trees have been recorded within the city`s tree cadastre and are managed by the municipal parks and garden department (Magistrat der Stadt Salzburg). During spring 2021 (April 12—June 2), birds were counted in 48 different sampling sites around the city (Fig. 1). Sampling sites were distributed throughout the city within the city border (Fig. 1) but restricted to the built-up areas of the city (Salzburg also contains large areas of arable land or forest within its city borders, see Fig. 1) and were selected in order to cover a large proportion of the available urbanization gradient as well as much of the city’s area, while limiting spatial autocorrelation. Specifically, we attempted to cover a large range of impervious surface covers across the city and selected sampling sites distributed across the city, confirming impervious surface cover visually in a 50-m radius when visiting the sites (see *Sampling site and local-scale habitat characteristics* below) and checking for accessibility to observers. The final range of impervious surface cover in the 50-m radius was 5–98% (Table S1). These sampling sites were situated in the city centre, small forests, public parks, private gardens, residential as well as industrial areas. We did not specifically select certain habitat types (e.g. ponds or lakes) but these were sometimes close to our sampling sites.

### Bird surveys

Fixed-radius bird point counts (Bibby et al. 2000) were carried out on working days between 06:30 AM and 9:00 AM under good weather conditions (avoiding windy days and precipitation) to coincide with peak singing activity. After a 5 min initiation period after the arrival on site, all bird individuals seen or heard within a 50-m radius were recorded for 10 min. This was done by the main observer standing in one spot and recording all the bird species heard or seen. At most, the observer moved around within the 50-m radius trying to identify a particular individual. Each sampling point was assessed twice 3–4 weeks apart, by two observers during the first round (Apr 12–30) to achieve maximum species cover and calibrate the main observer and by one observer (the same person that was always present in the first round) during the second round (May 4-June 2). Counts were combined across both observation rounds. Birds flying over the sampling radius were noted down but excluded afterwards from the analysis (e.g., swifts flying overhead with no apparent relationship with the sampling site). If more than one individual of the same species was unequivocally seen or heard at the same time, the number of individuals was noted as the maximum abundance. The bird species that were found were classified according to their original habitat (woodland, forest, grassland, human-modified, rock, shrubland or wetland habitat) based on information from the AVONET dataset (Tobias et al. 2022).

### Sampling site and local-scale habitat characteristics

Building cover (in %) was calculated in QGIS (version 3.22.11) based on OSM data provided by an inbuilt tool in QGIS ("QuickOSM") for a 50-m radius around each sampling point. Other QGIS-based variables were not included at this scale because we recorded variables in the field that we considered more informative. These sampling site-scale habitat characteristics were assessed once per sampling site between May 12 and June 2, 2021, within a 50-m radius around each sampling point. Tree canopy cover (in %) was estimated visually by the observer from the ground within this radius. Similarly, ground cover was estimated separately (in %) from the ground in different classes: shrub cover, impervious surface, grass cover and other cover. Shrub cover included bushes, woody plants, and trees with DBH of less than 20 cm. As impervious surface we considered roads, houses, buildings, and sealed surfaces of any kind. Grass cover included fields and meadows with herbaceous plants and lawns. Other kinds of ground cover were soil, gravel, water and rocks.

We counted all trees with a diameter at breast height (DBH) of more than 20 cm in the 50-m radius. In addition, to assess the number of mature trees, we also counted trees with a DBH of more than 50 cm separately. All tree species (DBH > 20 cm) found within 50 m around the sampling point were recorded. If it was not possible to identify the species, the tree was categorized as its genus (e.g., *Malus* sp.). Tree species were classified (based on Pilsl et al. 2008) as “native” to Salzburg or “exotic” (domesticated species or varieties and species not native to Salzburg, but partly native to other parts of Austria or Central Europe). Furthermore, we recorded the number of bird nest boxes in the 50-m radius.

We also conducted analyses with independent variables that were assessed at larger scales, namely 200 m and 500 m around the sampling sites, in QGIS. Specifically, we measured the mean impervious density (in %) based on Impervious Density raster data provided by Copernicus (10.2909/3bf542bd-eebd-4d73-b53c-a0243f2ed862), tree cover density (in %) based on a Copernicus raster dataset (10.2909/486f77da-d605-423e-93a9-680760ab6791), the area of street trees (in %) based on the Copernicus street tree layer (10.2909/205691b3-7ae9-41dd-abf1-1fbf60d72c8c), building cover (in %) based on OSM data calculated by an inbuilt tool in QGIS ("Quick OSM"), the relative area (in %) of the land-use types"Arable Land (annual crops)","Forests","Green urban area and"Pastures" based on Functional Urban Areas by Copernicus Urban Atlas data (10.2909/fb4dffa1-6ceb-4cc0-8372-1ed354c285e6).

### Statistical analysis

Data analysis was done in R version 4.3.1 (R Core Team 2023). First, we conducted Pearson correlation analyses between our independent variables at the three scales of the analyses (50 m, 200 m and 500 m). Of those variables that were highly correlated (correlation coefficient R > 0.6), the one with no other strong correlations was retained and the other one was removed. One sampling point was excluded from the models due to missing values in the independent variables.

To analyse relationships between independent variables and the bird community we used linear models with bird species richness as the response variable after visual inspection of the assumptions of homoscedasticity and normal distribution of the residuals. To analyse relationships with bird community composition, we ran a permutational multivariate analysis of variance (permanova) based on Sørensen dissimilarity (presence/absence) using the adonis2 function in package vegan (Oksanen et al. 2022) in R with 999 permutations. A Non-metrical Multidimensional Scaling (NMDS) plot based on Sørensen dissimilarity was used to graphically illustrate the association between the bird community and local habitat characteristics.

Furthermore, we analyzed the response of specific bird species (presence/absence) to habitat characteristics at sampling sites using generalized linear models with binomial error distribution. For consistency, the same independent variables were used as in the bird species richness model. We selected those species that occurred at intermediate frequencies. To analyse the relation between independent variables and the tree community, we used the same approaches as for the bird community.

## Results

In the tree communities within the 48 sampling sites throughout the city of Salzburg we found a total of 119 species of trees, 47 of which were native species and 72 were exotic species or domesticated varieties (Table S1). Most frequently occurred *Acer platanoides* (Norway maple, 23 sampling sites), *Carpinus betulus* (European hornbeam, 22 sampling sites), *Acer pseudoplatanus* (Sycamore maple) and *Fraxinus excelsior* (European ash, 21 sampling sites each), all of which are native species. The average tree species richness per sampling site was 11.6 ± 0.8 species (mean ± SE). We recorded a total of 39 bird species at the 48 sampling sites (Table S1). Some species were very common and recorded at most sampling sites, such as the Great tit (*Parus major*, 46 sites), the Common blackbird (*Turdus merula*, 45 sites) and the Eurasian blackcap (*Sylvia atricapilla*, 40 sites). Several species were only recorded once such as the Common buzzard (*Buteo buteo*), Common redstart (*Phoenicurus phoenicurus*), Common firecrest (*Regulus ignicapilla*) and the Eurasian siskin (*Spinus spinus*). The average bird species richness per sampling site was 9.9 ± 0.4 species.

The correlation analyses of the independent variables in the analysis of the bird communities (Figs. S1−3) left the following independent variables at the 50-m scale: building cover, grass cover, number of trees, number of old trees, number of exotic tree species, number of native tree species, and number of nest boxes. At the 200-m scale, we retained the following variables: arable land, building cover, forest, green urban area and pastures. In addition, the following sampling site-scale variables also used at the 50-m scale were retained: grass cover, number of trees, number of old trees, number of exotic tree species, number of native tree species and number of nest boxes. At the 500-m scale, we used the following variables: arable land, building cover, forest and green urban area. In addition, the sampling site-scale variables grass cover, number of trees, number of old trees, number of tree species and number of nest boxes were used in this analysis. For the analysis of tree communities, we retained the same independent variables for reasons of consistency but removed the number of tree species (since this is the response in this analysis) and the number of nest boxes from the list of independent variables.

**Fig. 2:**
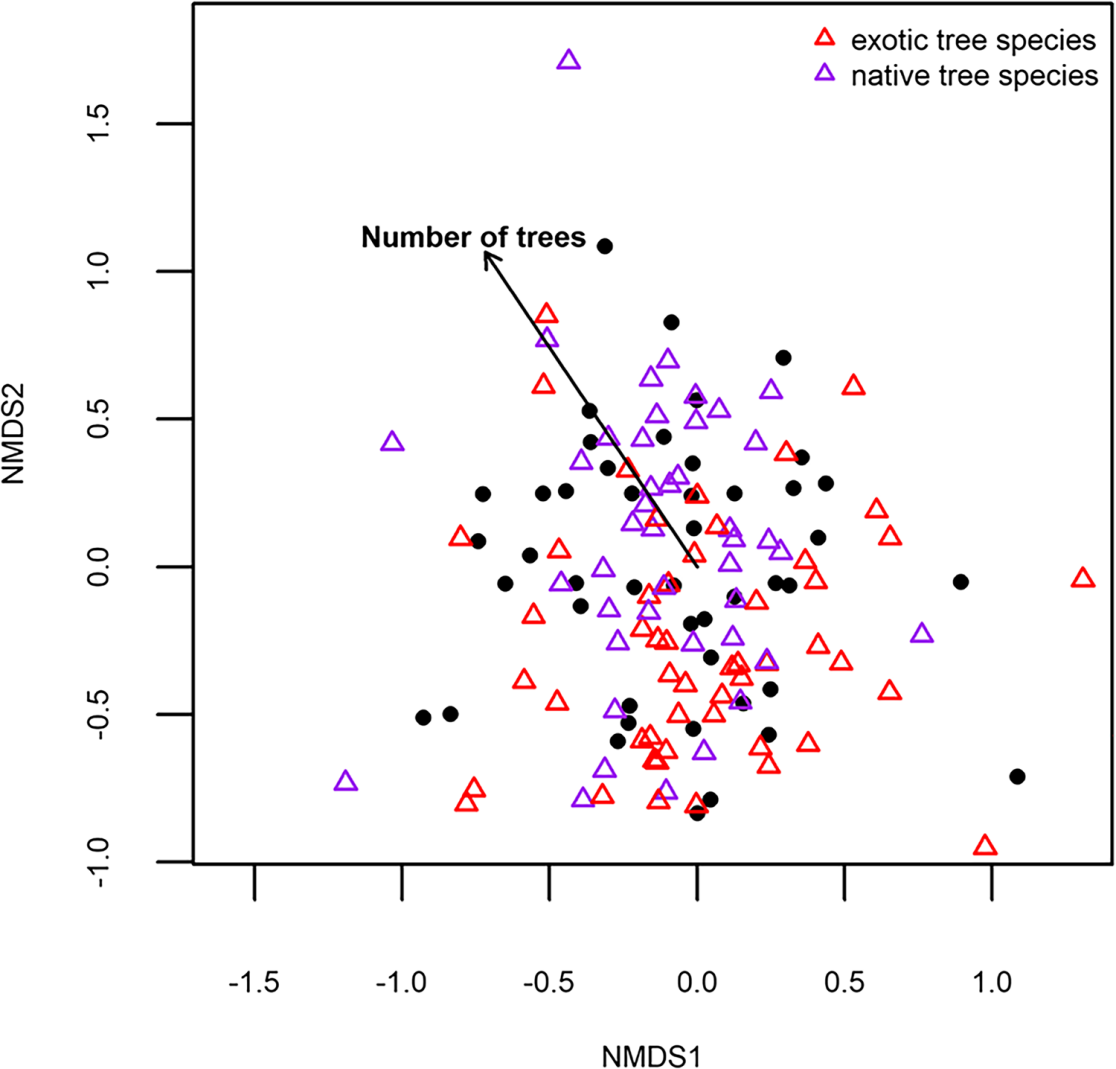
Non-metric multidimensional scaling (NMDS) plot of tree community composition at the different sampling sites (black dots) in Salzburg (Sørensen dissimilarity, stress = 0.15, k = 3). Exotic tree species or domesticated varieties are shown as red triangles, native tree species are shown as purple triangles. The number of trees recorded at a 50-m scale was the only environmental variable that had a significant influence on tree composition in the permanova (Table 1) and is plotted as an arrow

The species richness of trees showed a tendency for a positive relationship with grass cover (Table 1) but was not influenced by any other of the tested sampling site-level habitat characteristics (Table 1). When the species richness of native tree species was considered separately, it was negatively influenced by building cover (F_1,42_ = 4.11, *P* = 0.0490) and positively by the number of trees (F_1,42_ = 4.48, *P* = 0.0403). Exotic tree species richness was not related to any of the tested independent variables. Tree community composition was influenced solely by the number of trees (Table 1, Fig. 2). In separate analyses of native or exotic species only, this relationship was not significant. At larger spatial scales, building cover was negatively related to tree species richness and had an influence on community composition (Supplementary Tables S1 and S2).

**Table 1:** Results from a linear model on tree species richness and a permanova on tree community composition, testing the influence of sampling site-scale habitat characteristics in a 50-m radius, namely building cover (%), grass cover (%), the number of trees and the number of old trees (> 50 cm DBH)

	Tree species richness					Tree community composition				
	Df	SS	MS	F	P	Df	SS	R^2^	F	P
Building cover	1	77.89	77.89	2.54	0.1187	1	0.40	0.03	1.32	0.1510
Grass cover	1	121.13	121.13	3.95	0.0535	1	0.37	0.03	1.21	0.2370
Number of trees	1	43.96	43.96	1.43	0.2381	1	0.57	0.04	1.88	**0.0220**
Number of old trees	1	1.27	1.27	0.04	0.8401	1	0.38	0.03	1.25	0.2140
Residuals	42	1289.15	30.69			42	12.49	0.88		

*Df* degrees of freedom, *SS* Sums of squares, *MS* Mean square. *P*-values < 0.05 are printed in bold

**Fig. 3:**
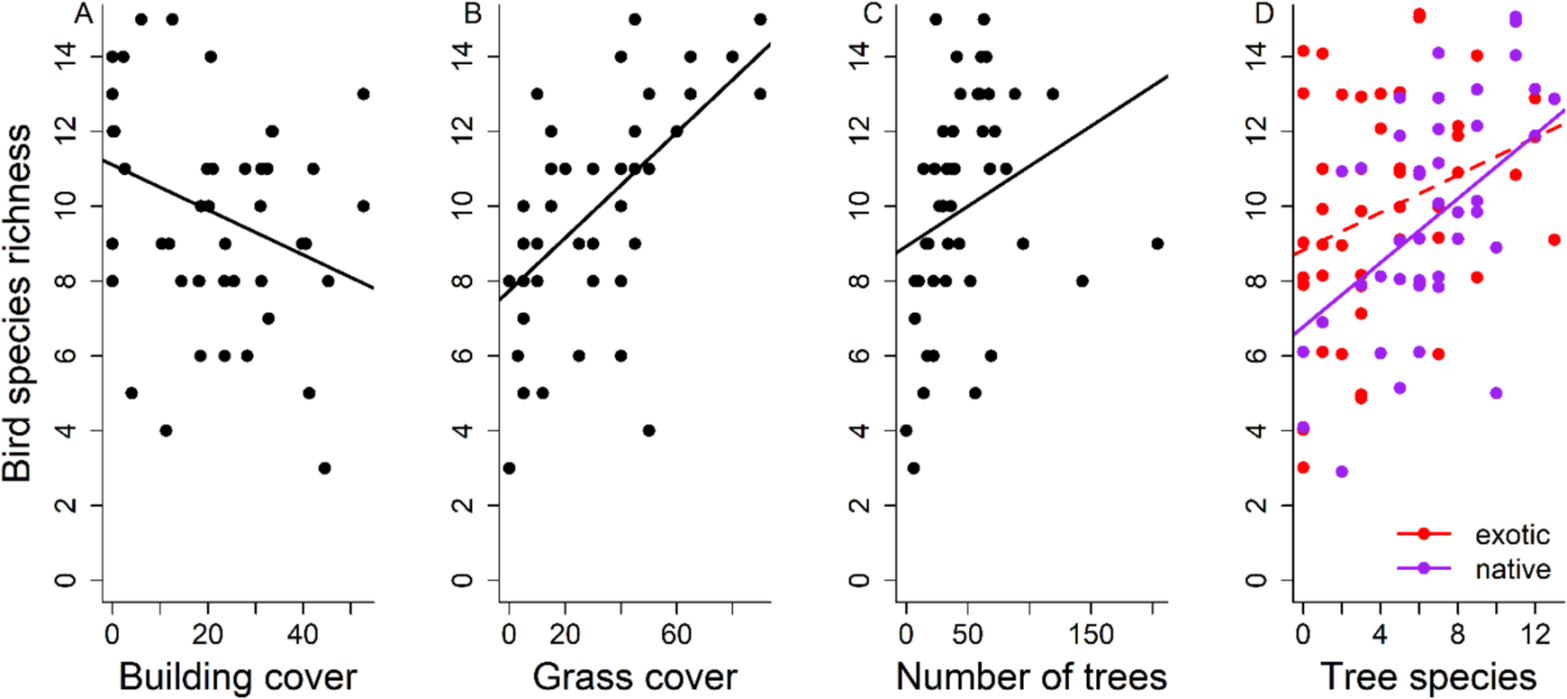
The relationship between **A**) building cover (in %), **B**) grass cover (in %), **C**) the number of trees and **D)** the number of tree species (the number of exotic tree species in red and native tree species in purple) in a 50-m radius and bird species richness in Salzburg. The relationship between the number of exotic tree species and bird species richness was not significant (Table 2) and is thus shown as a dashed line

**Fig. 4:**
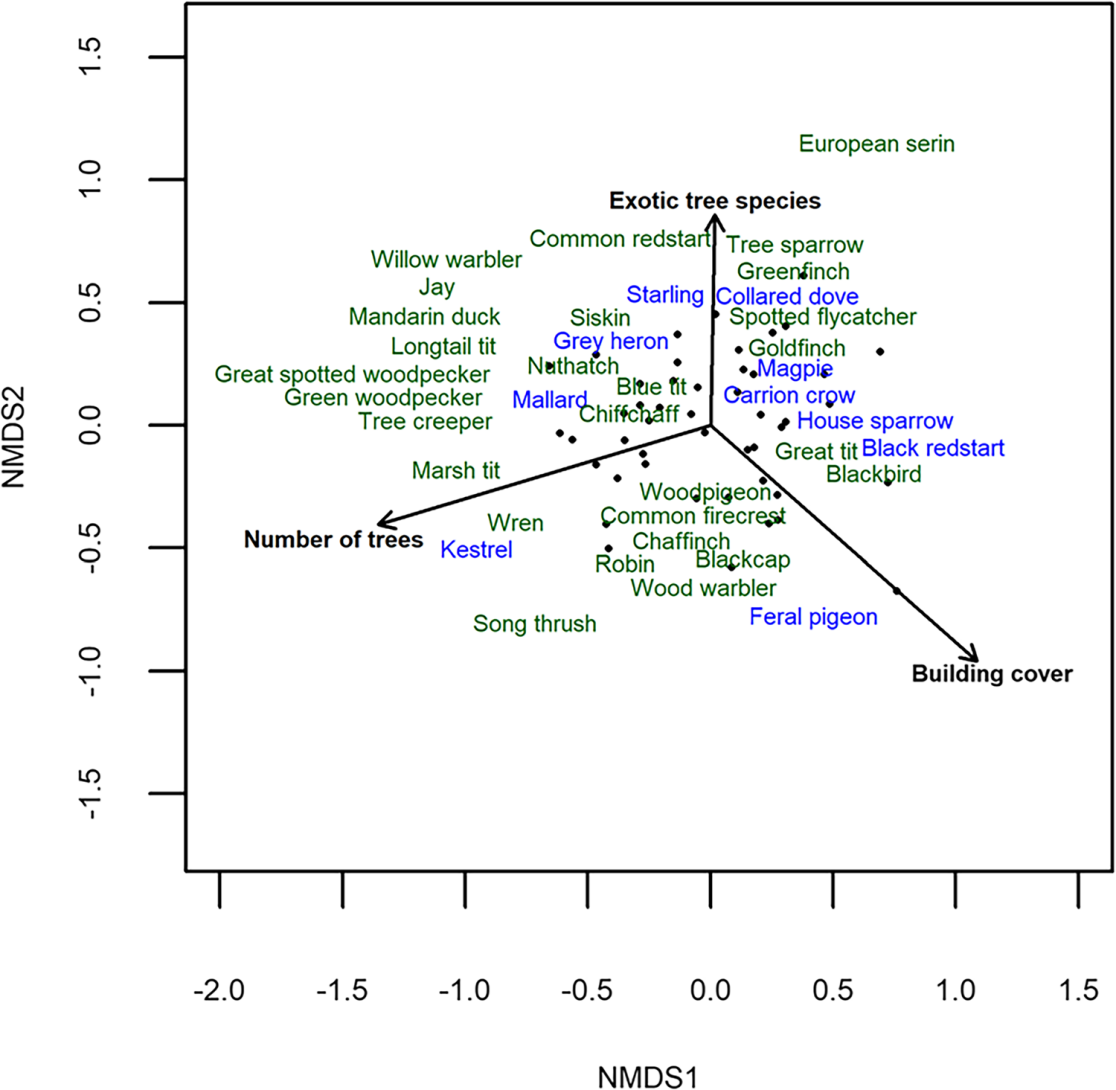
Non-metric multidimensional scaling (NMDS) plot of bird community composition at the different sampling sites (black dots) in Salzburg (Sørensen dissimilarity, stress = 0.15, k = 3). Species with woodlands and forests as original habitats are coloured in green, those with other habitats (grassland, human-modified, rock, shrubland and wetland habitats) are coloured in blue. Bird species names were moved slightly after plotting to avoid overlap. The environmental variables that had a significant relationship with bird composition in the permanova (Table 1) are plotted as arrows

Species richness of birds decreased with building cover in a 50-m radius around the recording point (Fig. 3A, Table 2), and increased with grass cover (Fig. 3B, Table 2), the number of trees (Fig. 3C, Table 2) and the total number of tree species (F_1,40_ = 7.56, *P* = 0.0089). However, when the number of exotic and native tree species were analysed separately, the relationship was only significant for the number of native tree species (Fig. 3D, Table 2). Bird community composition in a 50-m radius around the sampling point was significantly influenced by building cover, by the number of trees, and by the total number of tree species (F_1,40_ = 3.19, *P* = 0.0060, Fig. 4, Table 2). Variables at larger spatial scales (200 m, 500 m) showed largely similar relationships with bird species richness and community composition (Figs. S4-S7, Supplementary Tables S3, S4): apart from building cover, no other large-scale variable, i.e., the amount of forest, arable land or urban green area, showed a relationship with bird species richness, while the effects of sampling site-scale variables partly remained. Bird community composition was additionally influenced by green urban area at the 200-m and 500-m scale.

**Table 2:** Results from a linear model on bird species richness and a permanova on bird community composition, testing the relationship between local habitat characteristics in a 50-m radius, namely building cover (%), grass cover (%), the number of trees, the number of old trees (> 50 cm DBH), the number of exotic tree species, the number of native tree species and the number of nest boxes (see text for details) and bird species richness and bird community composition

	Bird species richness					Bird community composition				
	Df	SS	MS	F	P	Df	SS	R^2^	F	P
Building cover	1	39.11	39.11	7.96	**0.0075**	1	0.25	0.05	2.71	**0.0160**
Grass cover	1	95.85	95.85	19.51	**0.0001**	1	0.19	0.04	2.07	0.0710
Number of trees	1	28.33	28.33	5.77	**0.0212**	1	0.38	0.08	4.13	**0.0020**
Number of old trees	1	0.03	0.03	0.01	0.9428	1	0.12	0.03	1.35	0.2550
Number of exotic tree species	1	11.68	11.68	2.38	0.1312	1	0.20	0.04	2.14	0.0590
Number of native tree species	1	32.82	32.82	6.68	**0.0136**	1	0.15	0.03	1.67	0.1510
Number of nest boxes	1	1.40	1.40	0.29	0.5961	1	0.05	0.01	0.57	0.7580
Residuals	39	191.59	4.913			39	3.568	0.727		

*Df* degrees of freedom, *SS* Sums of squares, *MS* Mean square. *P*-values < 0.05 are printed in bold

**Fig. 5:**
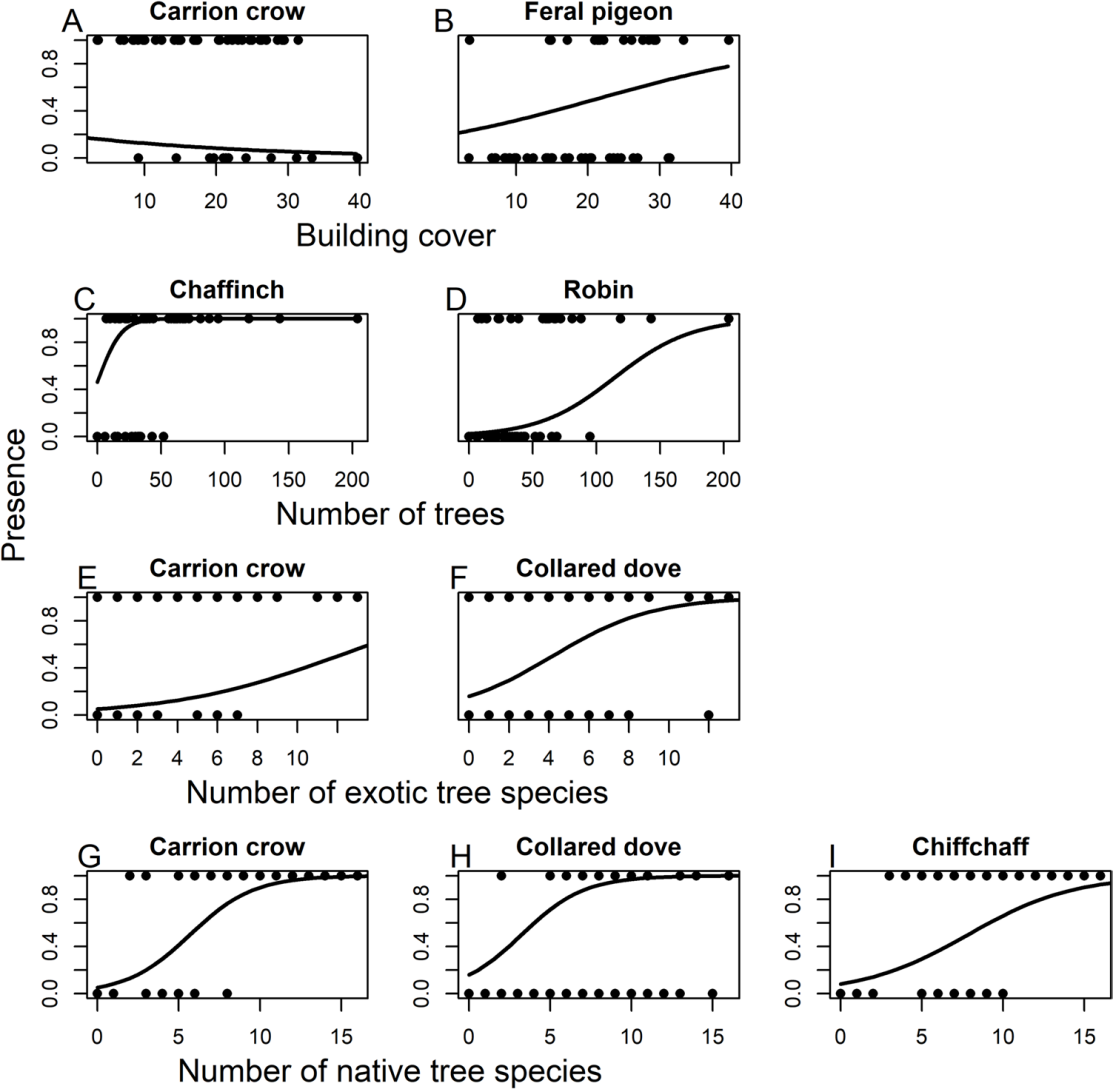
The relationship between **A**, **B**) building cover (in %), **C**, **D**) the number of trees, **E**–**F**) the number of exotic tree species, **G**-**I**) the number of native tree species in a 50-m radius around the recording point and the presence of specific bird species in Salzburg

We also assessed sampling site-level habitat preferences of specific bird species. Because of the frequent occurrence of only one individual per species in a sampling site, abundance data was not considered but presence-absence only. For this analysis, we selected those species that occurred at intermediate frequencies (i.e., from 36 sampling sites (Carrion crow) to 12 sampling sites (Starling)). The Carrion crow responded negatively to increasing building cover in a 50-m radius around the recording point (Fig. 5A, Table S5), the Feral pigeon positively (Fig. 5B). Chaffinch and Robin increased in frequency with increasing number of trees (Fig. 5C, D), the Carrion crow and Collared dove increased in frequency with an increasing number of exotic tree species (Fig. 5E, F) and Carrion crow, Collared dove and Chiffchaff increased with an increasing number of native tree species (Fig. 5G-I). The occurrence of Greenfinch, Black redstart and European starling was not dependent on any of the sampling site habitat characteristics (Table S5).

## Discussion

Most studies to date have assessed urban bird diversity within the green spaces of a city. Here, we show that birds can be found in all parts of the city, also outside of green spaces, but that species richness and composition vary depending on building cover, grass cover, and the number and diversity of trees. We found a large diversity of trees, both native and exotic, and tree species composition was influenced by the number of trees in a sampling site. While a lower species richness of native trees was found in sampling sites with high building cover, such a relationship was not found for exotic trees. Importantly, the relationship between trees and bird species richness was driven by the number of native trees, not exotic ones. In agreement with previous studies, we found that for bird species richness sampling site characteristics, i.e., grass cover and the number of native tree species, were more important than land-cover types at larger scales around sampling sites. An exception was the amount of building cover, which showed a negative relationship with bird species richness at all scales. Bird species composition was influenced by building cover and the number of trees in a sampling site. At larger scales bird species composition in a sampling site was related to building cover and urban green area, but less so to sampling site characteristics, except the number of trees in a sampling site when the largest scale (500 m) was considered. Three bird species occurred in almost every sampling site and at the species level, the occurrence of different species was associated with different sampling site characteristics. The Feral pigeon was the only species positively related to building density.

In agreement with previous studies, we found that urban bird species richness strongly depended on the greenness of a sampling site, i.e., the amount of grass and trees, and that the presence of these green characteristics is important at the smallest spatial scale (i.e., sampling site-scale, in our case 50 m) (Shwartz et al. 2013; Belaire et al. 2014; Mühlbauer et al. 2021; Fairbairn et al. 2024). The size of urban green spaces has been repeatedly shown to positively affect the presence of birds (Beninde et al. 2015; Kang et al. 2015), which corresponds with our finding that a higher amount of grass cover is associated with a higher number of different species in a sampling site. Furthermore, tree species richness was an important positive determinant of bird species richness in a sampling site, similar to studies in urban green spaces (Ferenc et al. 2014; Paker et al. 2014; da Silva et al. 2021; Kaushik et al. 2022). Different trees with different structural properties meet the habitat needs, e.g. food, nesting sites, and cover, of different bird species, thus contributing to the occurrence of a variety of birds with different functional traits (Nava-Díaz et al. 2020). In our study, the positive relationship with tree species richness was mainly driven by the number of native tree species, while the number of exotic tree species was not related to bird species richness, which matches findings from urban office developments (Dyson 2020). Accordingly, previous studies have found that the presence of native plants and trees in urban green spaces and streetscapes determines the richness and abundance of native birds (Burghardt et al. 2009; Ikin et al. 2013; Threlfall et al. 2016; Wood and Esaian 2020; Berthon et al. 2021), while exotic trees are favored by exotic birds (Paker et al. 2014). Exotic plants support fewer arthropods and thus provide insufficient resources for native bird species during breeding (Brändle et al. 2008; Jensen et al. 2021). In contrast to other studies, we did not find an effect of old (large) trees on bird species richness (Stagoll et al. 2012; Ferenc et al. 2014). A potential reason for this might be that we chose a relatively small DBH (> 50 cm). Trees with a larger DBH offer greater structural complexity (holes, structured bark) for foraging and breeding than smaller trees and thus have a larger effect on the inclusion of distinct species. We did not include shrub cover in our final analyses as it correlated positively with the number of trees at our sampling sites. However, for some species, shrub cover may be more important than tree cover or tree density as shrubs also provide cover close to the ground (Mühlbauer et al. 2024).

Although the tree species recorded most frequently in our study were all native, overall tree species richness was higher for exotic trees. Furthermore, in sampling sites with high building cover native tree species richness decreased, while exotic tree species richness was unaffected. High proportions of exotic plant species have been found across cities worldwide (Aronson et al. 2014; Jiao et al. 2021) and non-native trees are often found in residential areas (Jiao et al. 2021) and urban parks (Nielsen et al. 2014). In residential areas, tree species diversity is influenced by the preferences of residents for ornamental trees, but also by the availability at tree nurseries and gardening centres (Avolio et al. 2018; Jiao et al. 2021), while trees in parks and street trees depend on decisions by municipalities. Although some exotic trees may be used by birds as foraging sites, most exotic species are not (Wood and Esaian 2020). Therefore, while trees in general can provide important ecosystem services within cities, such as cooling and removal of emissions (Scholz et al. 2018), native trees also benefit local biodiversity and thus, by planting native trees, especially also in areas with high building cover, urban communities can potentiate positive ecosystem services of urban trees (Luck et al. 2011).

Similarly to a recent study within the city of Hamburg, where the amount of sealed surface was found to have a strong negative effect on bird diversity (Hastedt and Tietze 2023), we found only few species in densely built-up areas, and these were mostly species whose original habitat is associated either with rocks or which are closely associated with humans. Thus, high density urban areas with little vegetation promote biotic homogenization and loss of evolutionary distinctiveness (Morelli et al. 2024). Nevertheless, a few specialist species persist, and as these mostly depend on buildings as breeding sites, their needs should be accounted for in new developments or restoration sites (Jokimäki et al. 2018). Most species in our study were common species that originated from forest and woodland habitats and showed a strong positive association with the number of trees in a sampling site at the community level. The three most common species recorded in almost every sampling site, the Great tit, the Common blackbird, and the Eurasian blackcap, have been recently suggested as indicators of high urban environmental quality representing urban sites with relatively high vegetation cover and heterogeneity (Morelli et al. 2021). Thus, the city of Salzburg appears to be relatively green, supporting breeding pairs of these indicator species in many sites across its area.

Except for building cover, we did not find relationships between land cover types at larger scales and bird species richness, confirming the dominating role of sampling site-scale characteristics on urban birds found in previous studies (Shwartz et al. 2013). However, although we found the strongest relationship between land-cover/vegetation and bird species richness at the sampling site scale, due to spatial autocorrelation at a larger scale, i.e. > 500 m, we did not consider a larger landscape-wide scale, at which other land-cover types, such as forest cover, might have proven important. The negative relationship between building cover and bird species richness is in accordance with previous studies, which have shown that high-density urban areas and housing cover negatively impact bird species richness within adjacent woodlands, urban green spaces, or residential areas (Canedoli et al. 2018; Sidemo-Holm et al. 2022; Humphrey et al. 2023). At the community level both building cover and the amount of urban green area at the local scale (200 m and 500 m radius) had an influence on bird species composition. Especially forest specialists vulnerable to fragmentation may be affected by high building cover in the surroundings of a sampling site (Ferenc et al. 2014). Previous studies have also shown a positive influence of the presence of water bodies on urban bird diversity (Xie et al. 2022). Here we did not include sites with large water bodies. However, the city of Salzburg does encompass major tracts of water, e.g. the river Salzach, several smaller streams and lakes. Including such sites probably would have increased the number of species recorded and should be considered in future studies.

Considering individual species, Carrion crow and Collared dove were more likely to occur where there were more tree species (both native and exotic) in a sampling site, while Chiffchaff were positively related to the number of native tree species only. Chaffinches and Robins were positively related to the number of trees. Carrion crows and Collared doves are highly synanthropic species, who rely to a great extent on the availability of anthropogenic food sources. Indeed, collared doves have colonized Western European cities relatively recently, e.g. they arrived in Austria in the beginning of the last century, are exclusively found within settlements (Romagosa and Mlodinow 2022), and thus largely lack a common evolutionary history with the local flora and fauna. Chiffchaff are small insectivores that mainly forage in the canopy of trees, which may explain their preference for sites with many native trees, which support many arthropods (Brändle et al. 2008). Eurasian chaffinches and European robins are originally forest birds. Sampling sites with many trees were often near-natural sites dominated by native trees and the preference of these two species for such sites might indicate their dependence on higher tree cover. Thus, in contrast to overall species richness, single species may profit less from high tree species richness. Interestingly, some species, such as the house sparrow, showed no relation with sampling site-scale characteristics or were so common that they were found in almost every sampling site (see above). This might indicate a relatively high tolerance of some urban species for high building density and that already very little vegetation may suffice for these species. House sparrows, for example, have been shown to prefer sites with only 20–30% vegetation cover (Moudrá et al. 2018).

Sites sampled in our study were distributed randomly across the city and thus were heterogeneous with respect to building and tree cover. We are confident that using the point count method we were able to assess the presence/absence of species, however, we probably underestimated the number of individuals for some species and therefore did not include abundances in the analyses. Other studies have shown that sampling site characteristics not only influence the occurrence of species, but also their abundances (Mühlbauer et al. 2021). In addition, we did not assess the effects of noise or light (ALAN) on bird communities in Salzburg, which may also affect species composition in urban areas (Morelli et al. 2023). Furthermore, we did not consider variation in building height, which might be interesting to include in future studies (Rogers 2022).

Our study confirms the value of urban green, and in particular native trees, for the diversity of birds within cities. Urban green can be effective anywhere in the city and does not have to be restricted to urban parks, cemeteries or remnant semi-natural urban areas, although larger green spaces have the greatest potential. We show that tree species composition was related to bird species richness. Thus, in urban areas, the promotion of a diverse community of native trees and plants can have a positive impact on urban bird diversity. Although building cover had a strong negative influence at all scales, our study suggests that within densely built-up areas, reduction of the amount of sealed surface, greening of urban structures (e.g. green roofs and walls) and the retainment and planting of native street trees can positively influence bird diversity. Urban green and birds have been shown to positively influence human wellbeing (Methorst et al. 2021), thus promotion of these measures has the potential to transform cities into liveable places for both animals and humans.

## Supplementary Information

Below is the link to the electronic supplementary material.

Supplementary file1 (XLSX 53.1 KB)

Supplementary file2 (DOCX 1.32 MB)

## Data Availability

All data supporting the findings of this study are available within the paper and its Supplementary Information (Table S1).
