# Supplementary material for: Bird community responses to urbanization and vegetation parameters across the city of Salzburg, Austria
Source: Urban Ecosyst. 2025 May 28;28(4):131. doi: 10.1007/s11252-025-01738-y (PMC12119745; doi:10.1007/s11252-025-01738-y)
Supplement: Supplementary file 2 — Supplementary file2 (DOCX 1.32 MB) [file 11252_2025_1738_MOESM2_ESM.docx]

**Bird community responses to urbanization and vegetation parameters across the city of Salzburg, Austria**

**Beate A. Apfelbeck^1,*^, Marina Navalpotro Buscail^1,2^, Anna Sommer^1^, Jana S. Petermann^1^**

^1^ Department of Environment and Biodiversity, University of Salzburg, Hellbrunnerstrasse 34, 5020 Salzburg, Austria

^2^ Ecology Department, Biology Faculty, University of Barcelona, Diagonal, 643, 08028 Barcelona, Spain

^*^ Corresponding author: [beateanna.apfelbeck@plus.ac.at](mailto:beateanna.apfelbeck@plus.ac.at), https://orcid.org/0000-0001-7605-7060

**Supplementary Material**

**
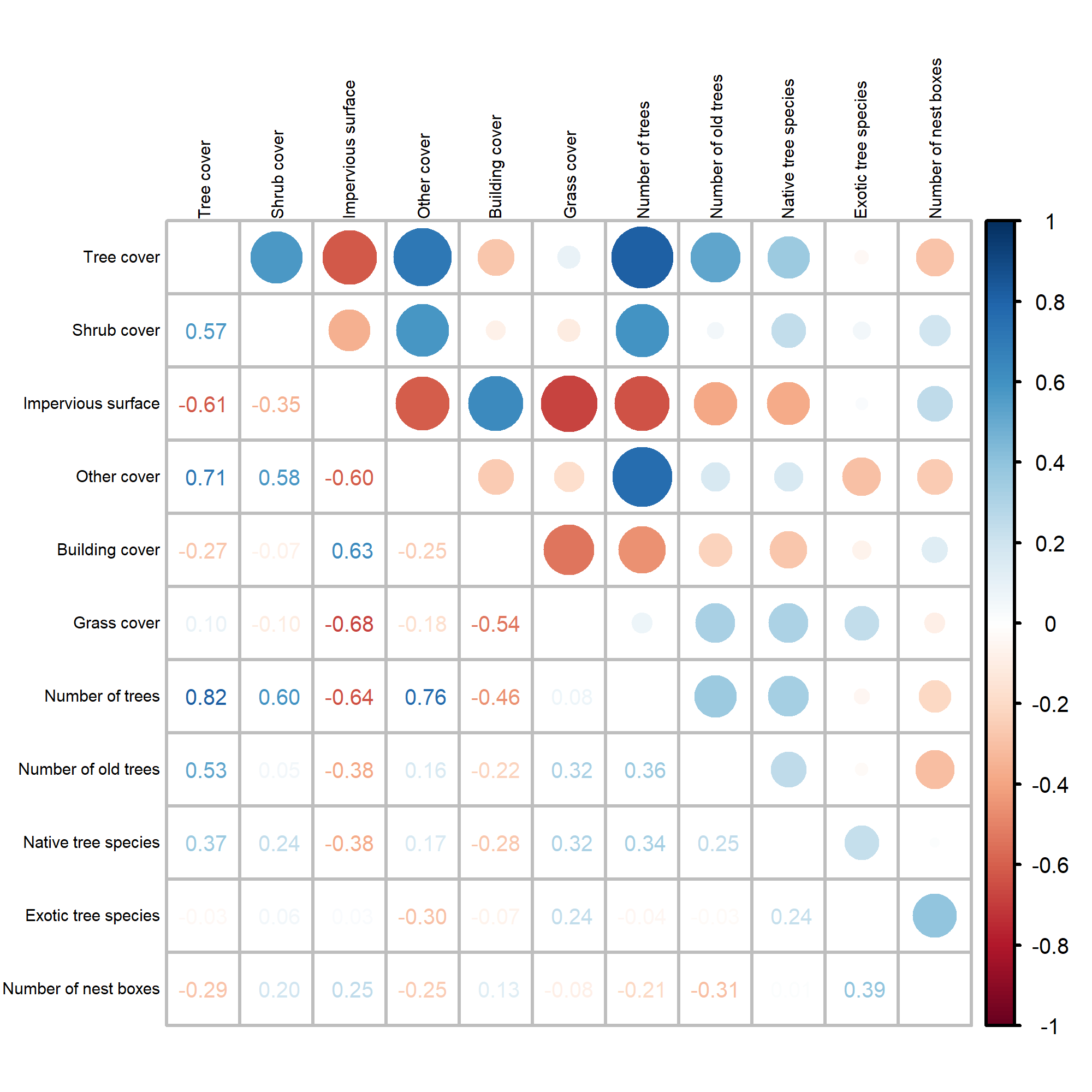
**

**Fig. S1:** Pearson correlation matrix between all sampling site habitat characteristics that were measured at a 50-m radius around the observation site.

**
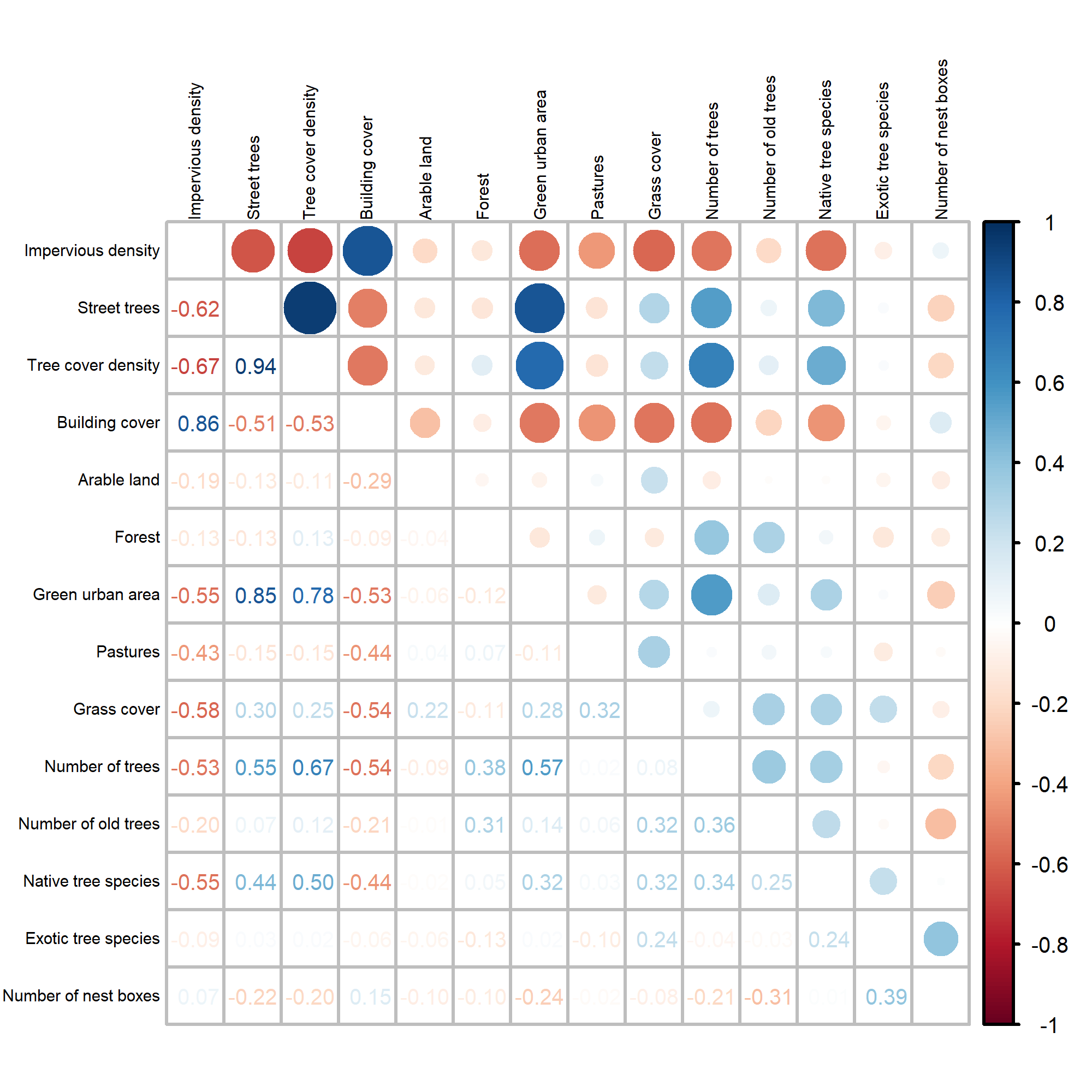
Fig. S2:** Pearson correlation matrix between environmental variables that were measured at a 200-m radius around the observation site and the sampling site habitat characteristics at 50-m radius that were included in the models at the 200-m scale.

**
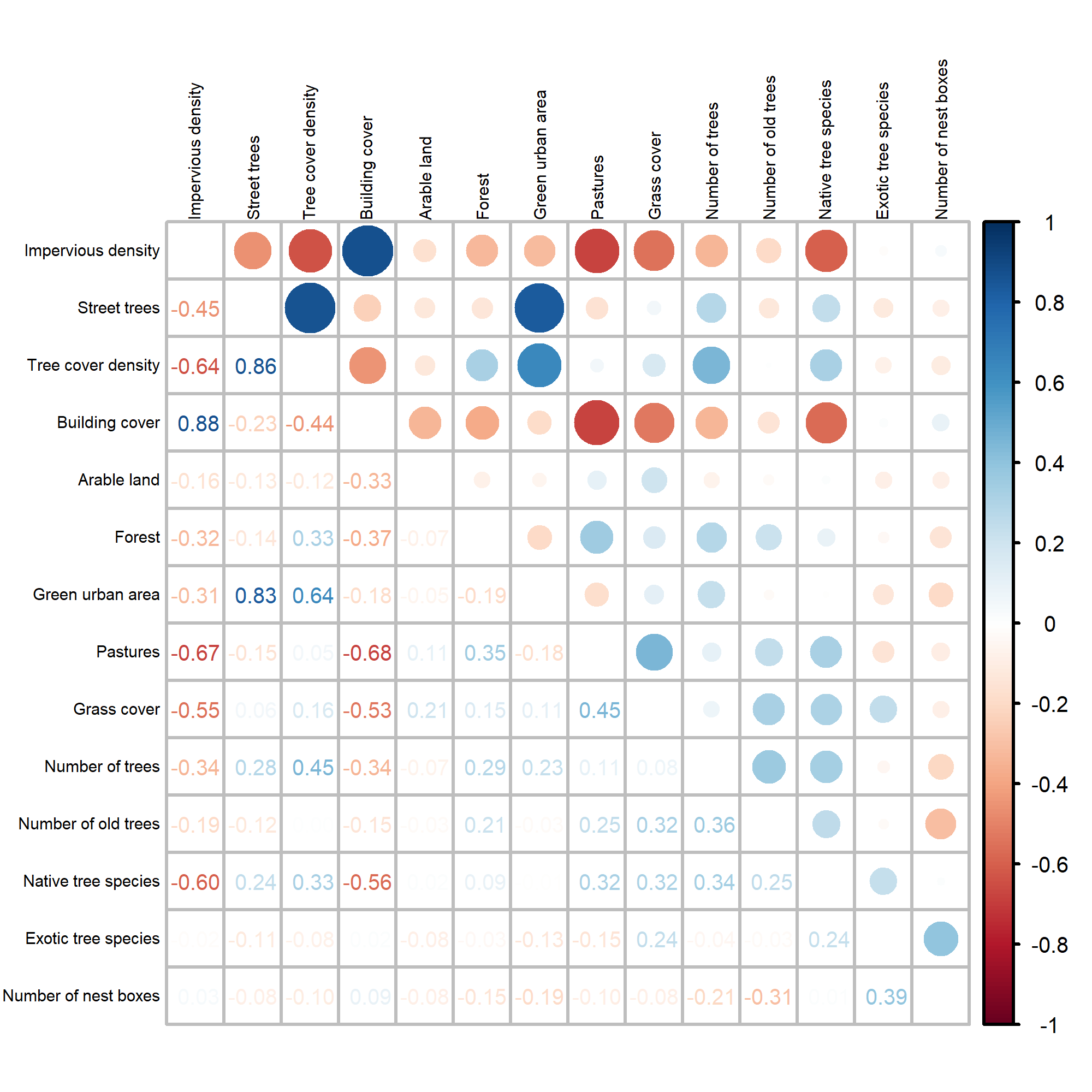
Fig. S3:** Pearson correlation matrix between environmental variables that were measured at a 500-m radius around the observation site and the sampling site habitat characteristics at 50-m radius that were included in the models at the 500-m scale.


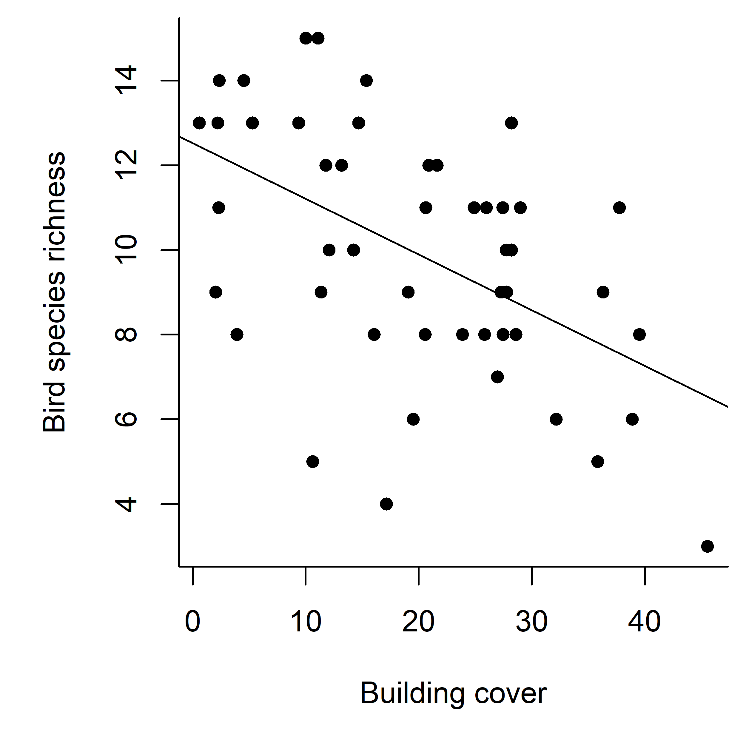


**Fig. S4**: The relationship between building cover (in %) in a 200-m radius and bird species richness in Salzburg.


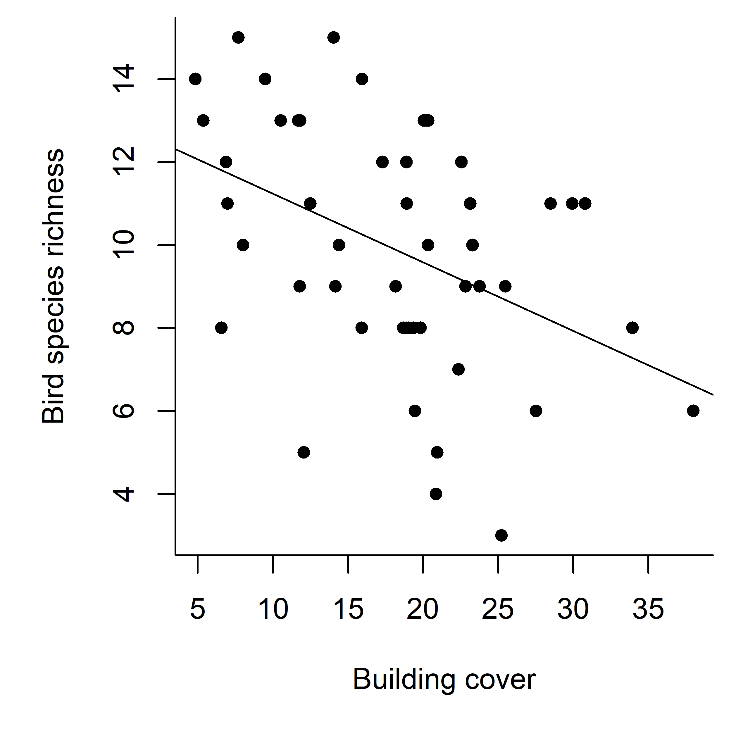


**Fig. S5**: The relationship between building cover (in %) in a 500-m radius and bird species richness in Salzburg.

**
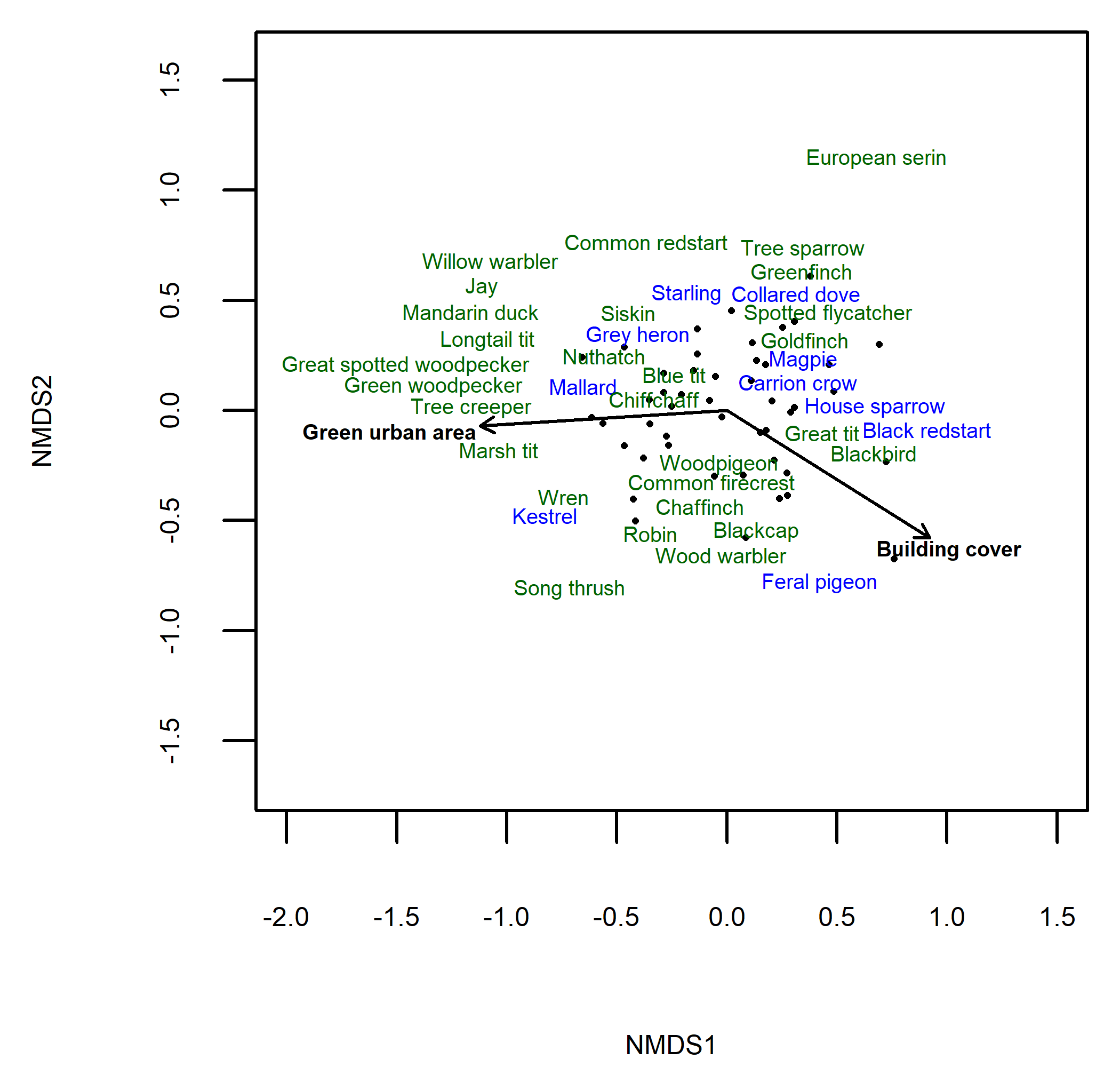
**

**Fig. S6**: Non-metric multidimensional scaling (NMDS) plot of bird community composition at the different sampling sites (black dots) in Salzburg (Sørensen dissimilarity, stress = 0.15, k = 3). Species with woodlands and forests as original habitats are coloured in green, those with other habitats (grassland, human-modified, rock, shrubland and wetland habitats) are coloured in blue. Bird species names were moved slightly after plotting to avoid overlap. The environmental variables that had a significant effect on bird composition in the permanova (Table S1) are plotted as arrows. Building cover and green urban area were measured in a 200-m radius around sites.


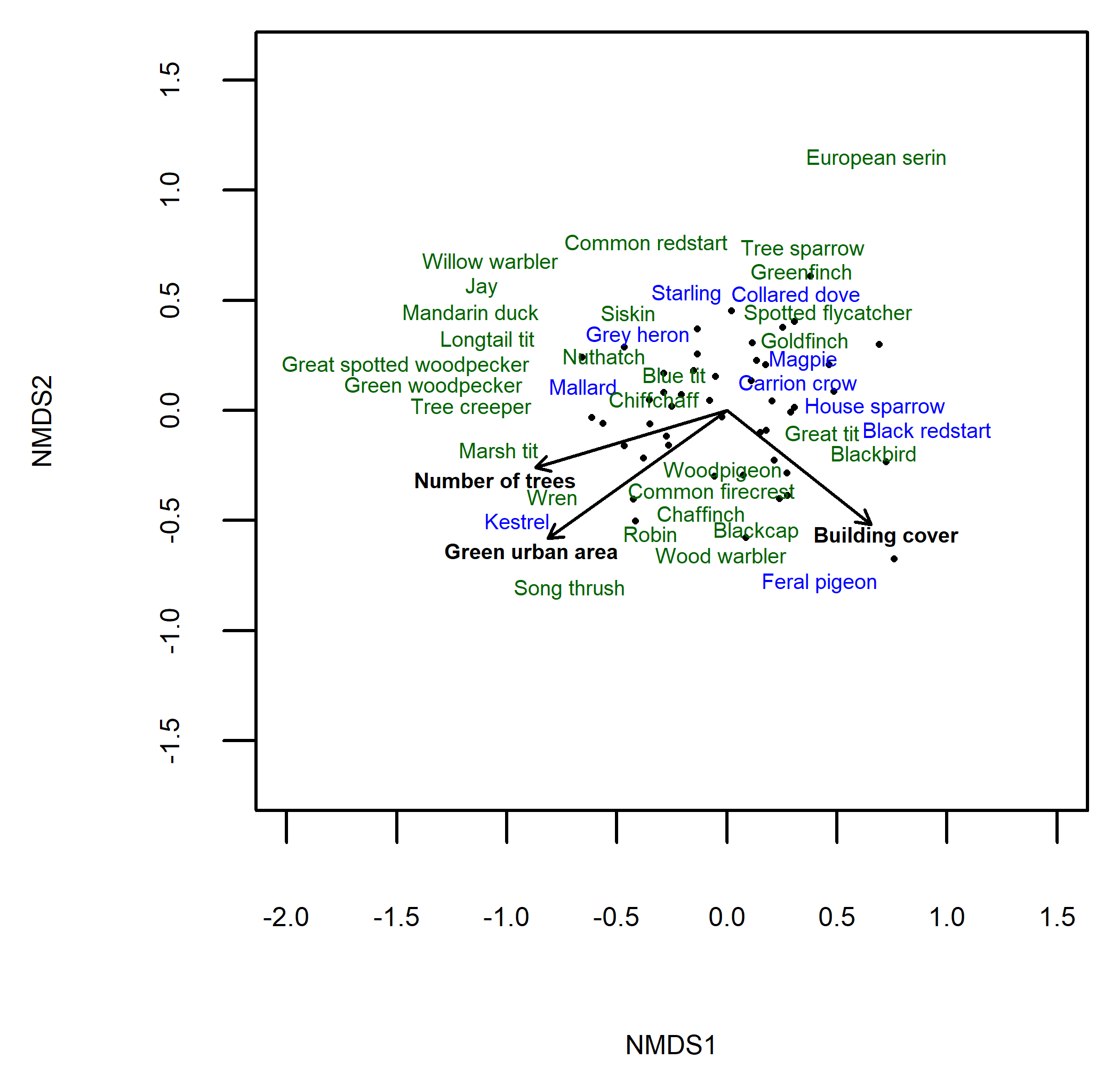


**Fig. S7**: Non-metric multidimensional scaling (NMDS) plot of bird community composition at the different sampling sites (black dots) in Salzburg (Sørensen dissimilarity, stress = 0.15, k = 3). Species with woodlands and forests as original habitats are coloured in green, those with other habitats (grassland, human-modified, rock, shrubland and wetland habitats) are coloured in blue. Bird species names were moved slightly after plotting to avoid overlap. The environmental variables that had a significant effect on bird composition in the permanova (Table S2) are plotted as arrows. Building cover and green urban area were measured in a 500-m radius around sites, the number of trees in a 50-m radius.

**Table S1:** Results from a linear model on tree species richness and a permanova on tree community composition, testing the influence of land cover variables at the scale of a 200-m radius around the sites: building cover (%), arable land (%), forests (%), green urban area (%), pasture (%), and of a number of variables at the 50-m scale which were recorded on the ground: grass cover (%), the number of trees and the number of old trees (> 50 cm DBH, see text for details). Df= degrees of freedom, SS= Sums of squares, MS= Mean square. P-values < 0.05 are printed in bold.

**Table S2:** Results from a linear model on tree species richness and a permanova on tree community composition, testing the influence of land cover variables at the scale of a 500-m radius around the sites: building cover (%), arable land (%), forests (%), green urban area (%), and of a number of variables at the 50-m scale which were recorded on the ground: grass cover (%), the number of trees, the number of old trees (> 50 cm DBH, see text for details). Pasture cover was not tested at the 500-m scale because it correlated negatively with building cover at this scale. Df= degrees of freedom, SS= Sums of squares, MS= Mean square. P-values < 0.05 are printed in bold.

**Table S3:** Results from a linear model on bird species richness and a permanova on bird community composition, testing the influence of land cover variables at the scale of a 200-m radius around the sites: building cover (%), arable land (%), forests (%), green urban area (%), pasture (%), and of a number of variables at the 50-m scale which were recorded on the ground: grass cover (%), the number of trees, the number of old trees (> 50 cm DBH), the number of exotic tree species, the number of native tree species and the number of nest boxes (see text for details). Df= degrees of freedom, SS= Sums of squares, MS= Mean square. P-values < 0.05 are printed in bold.

**Table S4:** Results from a linear model on bird species richness and a permanova on bird community composition, testing the influence of land cover variables at the scale of a 500-m radius around the sites: building cover (%), arable land (%), forests (%), green urban area (%), and of a number of variables at the 50-m scale which were recorded on the ground: grass cover (%), the number of trees, the number of old trees (> 50 cm DBH), the number of exotic tree species, the number of native tree species and the number of nest boxes (see text for details). Pasture cover was not tested at the 500-m scale because it correlated negatively with building cover at this scale. Df= degrees of freedom, SS= Sums of squares, MS= Mean square. P-values < 0.05 are printed in bold.

**Table S5:** Results from generalized linear models on the presence-absence of specific bird species, testing the effects of sampling site habitat characteristics in a 50-m radius, namely building cover (%), grass cover (%), the number of trees, the number of old trees (> 50 cm DBH), the number of exotic tree species, the number of native tree species and the number of nest boxes (see text for details). Only P-values are given, with those P-values < 0.05 printed in bold.
